# Discrimination of Microplastics and Phytoplankton Using Impedance Cytometry

**DOI:** 10.1021/acssensors.4c01353

**Published:** 2024-08-14

**Authors:** Jonathan T. Butement, Xiang Wang, Fabrizio Siracusa, Emily Miller, Katsiaryna Pabortsava, Matthew Mowlem, Daniel Spencer, Hywel Morgan

**Affiliations:** † School of Electronics and Computer Science, University of Southampton, Southampton SO17 1BJ, United Kingdom; ‡ National Oceanography Centre, Southampton SO14 3ZH, United Kingdom

**Keywords:** microplastics, phytoplankton, impedance cytometry, impedance spectroscopy, machine learning, lab-on-a-chip

## Abstract

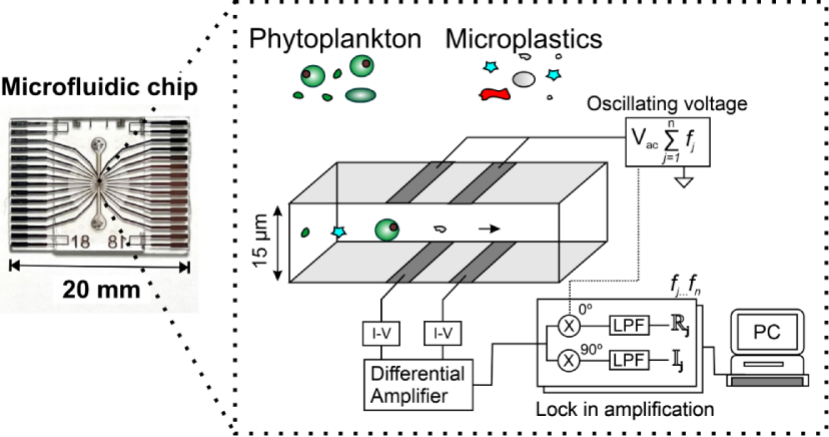

Both microplastics and phytoplankton are found together in the ocean as suspended microparticles. There is a need for deployable technologies that can identify, size, and count these particles at high throughput to monitor plankton community structure and microplastic pollution levels. In situ analysis is particularly desirable as it avoids the problems associated with sample storage, processing, and degradation. Current technologies for phytoplankton and microplastic analysis are limited in their capability by specificity, throughput, or lack of deployability. Little attention has been paid to the smallest size fraction of microplastics and phytoplankton below 10 μm in diameter, which are in high abundance. Impedance cytometry is a technique that uses microfluidic chips with integrated microelectrodes to measure the electrical impedance of individual particles. Here, we present an impedance cytometer that can discriminate and count microplastics sampled directly from a mixture of phytoplankton in a seawater-like medium in the 1.5–10 μm size range. A simple machine learning algorithm was used to classify microplastic particles based on dual-frequency impedance measurements of particle size (at 1 MHz) and cell internal electrical composition (at 500 MHz). The technique shows promise for marine deployment, as the chip is sensitive, rugged, and mass producible.

Microplastics are particles and fibers of plastic of millimeter scale and below. Microplastic pollution in the ocean is a growing concern as there is potential for wide ranging negative effects on the marine ecosystem and human health.^1,2^ Estimates of the levels of microplastic pollution vary and depend on the sampling and analysis technique used. There is estimated to be up to 21 million tons of the three most common microplastics polyethylene, polypropylene, and polystyrene in the top 200 m of the Atlantic alone within the size range of 32–651 μm.^3^ Little is known about microplastics smaller than 25 μm in diameter due to a lack of available techniques capable of measuring microplastics of this size range.

Microplastics are found suspended in seawater among a variety of other microscopic particle types including inorganic particles such as sand and silt, zooplankton, eggs, and phytoplankton, which are in particularly high abundance. Phytoplankton are a diverse set of microscopic photosynthetic organisms which form the base level of the food web in the ocean and are responsible for almost 50% of primary production on earth.^4^ The health, composition, and quantity of phytoplankton in the ocean have far-reaching consequences for the global carbon cycle and ecosystems. Currently, there is concern about the changing composition of phytoplankton communities in response to human-induced climate change^5^ showing an increased rate of turnover and an unstable community structure.^6^

There is a need for technologies which can identify, count, and size microplastics and phytoplankton in seawater. The ability for such technologies to perform in situ analysis is particularly desirable to allow deployment on automated vehicles, which are increasingly being used for oceanic surveys. Currently, there are a range of analytical techniques used to analyze microplastics and phytoplankton. Standard microscopy allows visual classification of particles and is affordable and accessible but has a low throughput and relies on user experience.^7^ Scanning electron microscopy allows identification of the smallest particles but has a very low throughput and is difficult to deploy in the field.^8^ Fourier transform infrared spectroscopy (FTIR) uses the mid-infrared absorption signature of plastics to identify material, typically collected on a filter. The technique is label-free and specific but is expensive and time-consuming. Imaging FTIR allows individual particles to be identified but is limited to particles above 10 μm in diameter.^9^ Raman spectroscopy also allows label-free identification of microplastics and can theoretically identify particles down to 1 μm in diameter.^10,11^ Raman spectroscopy can be used as a complementary technique to FTIR but suffers from the same drawbacks of low throughput and limited deployability when in a conventional microscopy format. Raman spectroscopy has been used in combination with holographic imaging in a deployable format to identify 3 mm plastic pellets suspended in water without the need for filter collection.^12^ Conventional flow cytometry offers an alternative method for analyzing particles from optical scattering and fluorescence signals. Thousands of individual particles can be analyzed per second by flowing them rapidly through a laser beam providing information on particle size and pigment content. Standard benchtop flow cytometers have been used onboard ship^13^ as well as specialized deployable cytometers.^14^ Phytoplankton are identified by intrinsic fluorescence from photosynthetic pigments, while microplastic identification relies on highly multidimensional analysis^15^ or prestaining with a fluorophore such as Nile red^16,17^ which is challenging in situ. Imaging flow cytometry has been widely used for identification and enumeration of phytoplankton in situ,^18^ but there are limited image analysis techniques available for microplastics without additional identification markers.^19^

Impedance cytometry is a potential technique that can be used for the analysis and quantification of microplastics and phytoplankton. Impedance cytometers measure the size and electrical properties of individual particles or cells,^20,21^ and this information can be used for discriminating microplastics from phytoplankton or potentially discriminating between individual species of phytoplankton.^22^ Chip-based impedance cytometry is advantageous for ocean sensing as it allows high-throughput, label-free single particle analysis and importantly can be easily miniaturized and ruggedized for the in-field analysis. It also avoids the need for bulky and complex sheath flow-based hydrodynamic focusing.^23^ Early work in the field demonstrated that impedance spectroscopy combined with fluorescence could size and classify selection phytoplankton.^24^ Sui et al. used multifrequency impedance cytometry to track cell health in response to changing medium salinity.^25^ de Bruijn et al. used impedance opacity measurements to discriminate between calcified and decalcified coccolithophores.^26^ Two-frequency impedance spectroscopy has also been used to quantify hundreds of microplastics and biological particles in fresh water in the 200–1000 μm size range.^22^ However, the smaller size range of phytoplankton and microplastics below 10 μm is more difficult to characterize by impedance cytometry. In addition, most techniques analyze the sample in low conductivity media, which limits direct sampling from the ocean.

In this work, we demonstrate a significant step toward using impedance cytometry to discriminate microplastics from phytoplankton directly in seawater. A novel impedance cytometry chip and a protocol are used to quantify microplastics in a mixed culture of phytoplankton using dual-frequency impedance measurements and simple machine learning algorithms. This method targets the yet uncharacterized size fraction of 1.5–10 μm particles in a full conductivity seawater medium (≈3 S/m). We also identify some of the challenges and limitations associated with using impedance to identify particles and discriminate between phytoplankton species in a complex mixture, such as seawater, with an example analysis of a sample of dock water.

## Device Design and Measurement Principle

**Figure 1:**
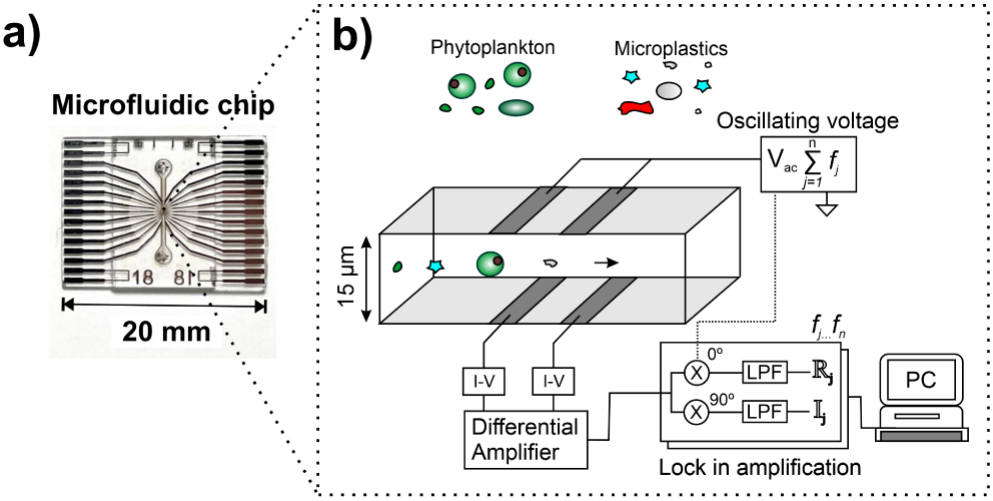
(a) Plan view photograph of a glass microfluidic chip. (b) Schematic cross section of a microfluidic channel showing the simplified measurement principle for multifrequency impedance cytometry.

The device design, fabrication, and measurement principle have been previously described in detail by Spencer and Morgan.^21^ The microfluidic chip is shown in Figure 1a, and the measurement concept for impedance cytometry is shown conceptually in Figure 1b. A microfluidic channel is formed from two glass wafers with a layer of patterned photoresist inbetween, with pairs of parallel facing microelectrodes on the roof and floor of the channel. Seawater containing particles flows through the microfluidic channel, and an AC voltage is applied to the electrodes. The electric current passing between opposing electrodes is measured differentially using a lock-in amplifier. A particle flowing along the channel between the electrodes disturbs the flow of electrical current, and the amplitude and the phase of the current change correspond to the complex impedance of the particle. The chips used in this work use nine pairs of parallel facing electrodes which produce a multipeak impedance signal as previously described in the literature.^27^ This allows the position and velocity of the particle in the channel to be measured which is then used to normalize the impedance signal and improve measurement precision. The impedance of a single particle at multiple discrete frequencies is measured simultaneously using a multichannel lock-in amplifier allowing for high-throughput single-cell impedance spectroscopy. Phytoplankton cells and microplastics exhibit distinct impedance spectra due to their structure, and this information can be used to classify particles. Specific frequencies can be used to probe distinct electrical properties of particles. In high conductivity media (seawater) at low frequencies (0.5–10 MHz), impedance is a direct measure of particle volume for both phytoplankton cells and microplastics. At intermediate frequencies (10–200 MHz), the impedance of cells is dominated by membrane capacitance, while at high frequencies (>200 MHz), the signal from a cell is dictated by internal conductivity and the electrical properties of any organelles. By comparison, microplastics are solid objects with a fixed value of electrical permittivity and conductivity.

High-performance lock-in amplifiers are capable of taking single-particle impedance measurements at 8 or more frequencies simultaneously over a broad frequency range; however, performing such multifrequency measurements on a marine deployment poses challenges. The drive voltage must be split between each channel, which lowers the signal-to-noise ratio; a high data transfer rate and large capacity data storage are required. Such multifrequency lock-in amplifiers are also expensive, bulky, and difficult to ruggedize. A simpler approach is to carefully select two optimal frequencies to classify particles. A low-frequency signal was used to size particles, and a high-frequency signal was used to identify differences in internal electrical properties.

## Materials and Methods

### Phytoplankton and Microplastic Samples

Monodisperse phytoplankton cultures of *Isochrysis galbana* (IG), *Chlorella vulgaris* (CV), *Porphyridium purpureum* (PP), and *Synechococcus sp.* (SY) were purchased from the Culture Collection of Algae and Protozoa (Oban, Scotland). The cultures were grown in an incubator at 18 °C under warm white (3000K) LED light at an irradiance of 1.6 mW/cm^2^ with a day/night cycle of 12/12 h. IG, CV, and PP were grown in the f/2 medium^28^ and SY in the L1 medium.^29^ The phytoplankton cultures contained predominantly one type of phytoplankton, as verified by visual inspection and size measurement under a microscope. All cultures showed some evidence of coliving bacteria or cyanobacteria but still contained a high proportion of the species of interest. Two-week-old cultures were used for the impedance cytometry experiments. Plastic calibration beads were used to simulate microplastics. A sample of microplastics was prepared by mixing a range of calibration beads made of both polystyrene (diameter 2,3,5 μm) and PMMA (diameter 8 μm) (Merck, Germany) in f/2 media.

### Impedance Cytometry

Microfluidic impedance cytometry chips were fabricated as described in detail elsewhere.^30,31^ In brief, platinum microelectrodes were first patterned onto 6 in. glass substrates. Microfluidic channels were patterned using a 15 μm thick negative photoresist (Perminex 2000, KayakuAM, USA) layer on the base substrate, which was aligned and bonded to the top substrate using a thermal bonder (EVG 620TB, EV group, Austria). An automated scriber was used to dice individual chips. Fluidic input and output access holes were drilled into the top substrate by laser machining (Epilogue Fusion edge 40W, Epilogue USA). The input and output fluidic tubing connections were made to the chip using a clamp-on tubing manifold shown in Figure S2. Electrical connections were made to the chip electrode pads by using contact spring pins. Samples were transferred into a syringe and immediately flowed through the microcytometer at 30 μL/min. Particle impedance was measured using a custom PCB front-end amplifier and a digital lock-in amplifier (UHFLI, Zurich instruments). A 4 V_pk-pk_ excitation signal was used, and data were recorded at 230 ksamples/s.

### Population-Averaged Impedance Spectra

The method for obtaining population-averaged impedance spectra using microfluidic cytometry has previously been described in detail.^21^ The population-averaged impedance spectrum of *I. galbana* in the f/2 medium was taken by measuring the average impedance for 10000 cells at incremental excitation frequencies between 250 kHz and 550 MHz. For each measurement, a reference impedance measurement was also made at 80 MHz from a population of 5 μm diameter polystyrene beads mixed in with the sample in order to track baseline drift. Fitting of a double-shell electrical model^32^ to the experimental data was performed by minimizing the mean square error between the analytical model and the experimental data using the MATLAB function “pattern search”.

### Dual-Frequency Impedance Cytometry of Monocultures and Mixed Samples

The impedance cytometry apparatus is shown in Figure S2. Dual-frequency impedance analysis of samples was conducted at 1 and 500 MHz simultaneously. Immediately before microcytometry analysis, all samples were filtered through a 40 μm diameter pore size cell strainer. To allow size calibration of each monodisperse sample, 2 μm diameter polystyrene calibration beads were mixed into each sample to produce a final concentration of 100 beads/μL. To acquire training data for KNN classification, the dual-frequency impedance of individual particles in monodisperse samples was recorded to build a training library with a minimum of 3000 particle signals for each class. The concentration of cells in each monodisperse sample was calculated from these impedance cytometry data by gating out cell populations. A mixed culture of phytoplankton and microplastics was prepared by mixing each monodisperse sample of microplastics: *I. galbana*: *C. vulgaris*: *Synechococcus*: *P. purpureum* stock samples, respectively, at an equal volumetric ratio. In the mixed culture, the final concentration of 2 μm beads was increased to 200 beads/μL to enhance the 2 μm bead peak for calibration. The conductivity of the mixed culture medium and monocultures was measured at 2.9 S/m on a Horiba LAQUAtwin conductivity meter.

### Machine Learning Classification of Microplastics and Phytoplankton

The KNN algorithm used two dimensions for classification: particle radius (cubed root of the real part of impedance measured at 1 MHz) and particle impedance phase at 500 MHz. Training data sets for each class were formed from the impedance cytometry data of each monodisperse sample. Training populations of cells or microplastics were manually gated from contour scatter plots based on expected particle sizes. All training data sets had the same number of data points, *n* = 3000. The KNN algorithm was written in MATLAB using the standard “fitknn” function with a standardized Euclidean distance metric, no weighting, exhaustive search method, and default cost matrix. The number of nearest neighbors used for the algorithm was 11 as this provided the minimum cross validation loss (Figure S1). KNN classification accuracy was evaluated by measuring the recovery rate for KNN classification of a mixed sample. The recovery rate is defined as the percentage ratio of KNN classified particle concentration against the known concentration of each particle class added to the mixed culture.

### Flow Cytometry of Dock Water

Dock water was collected from the dock front at the National Oceanography Centre, Southampton at high tide on 13/06/23. The sample was filtered using a 10 μm mesh cell strainer. The impedance cytometer was used to analyze a 120 μL sample using the standard protocol described in Dual-Frequency Impedance Cytometry of Monocultures and Mixed Samples. The KNN algorithm was trained as a binary algorithm to sort between microplastics and biological particles. The phytoplankton training data set was formed by pooling the training data for all of the phytoplankton monocultures in a single data set representing biological particles. The microplastic training set consisted of a mixture of 2, 3, and 5 μm polystyrene together with 8 μm PMMA particles. The KNN algorithm process was otherwise identical to that used for analysis of the mixed culture. 600 μL of the dock water sample was also analyzed on a conventional flow cytometer (Attune Nxt, Thermo Fisher Scientific, US) to compare total particle counts. Particle recognition was triggered from forward scatter with the trigger threshold set to produce no triggers when a 0.2 μm filtered sample of dock water was run through the cytometer.

## Results and Discussion

### Impedance Spectroscopy of Phytoplankton

**Figure 2:**
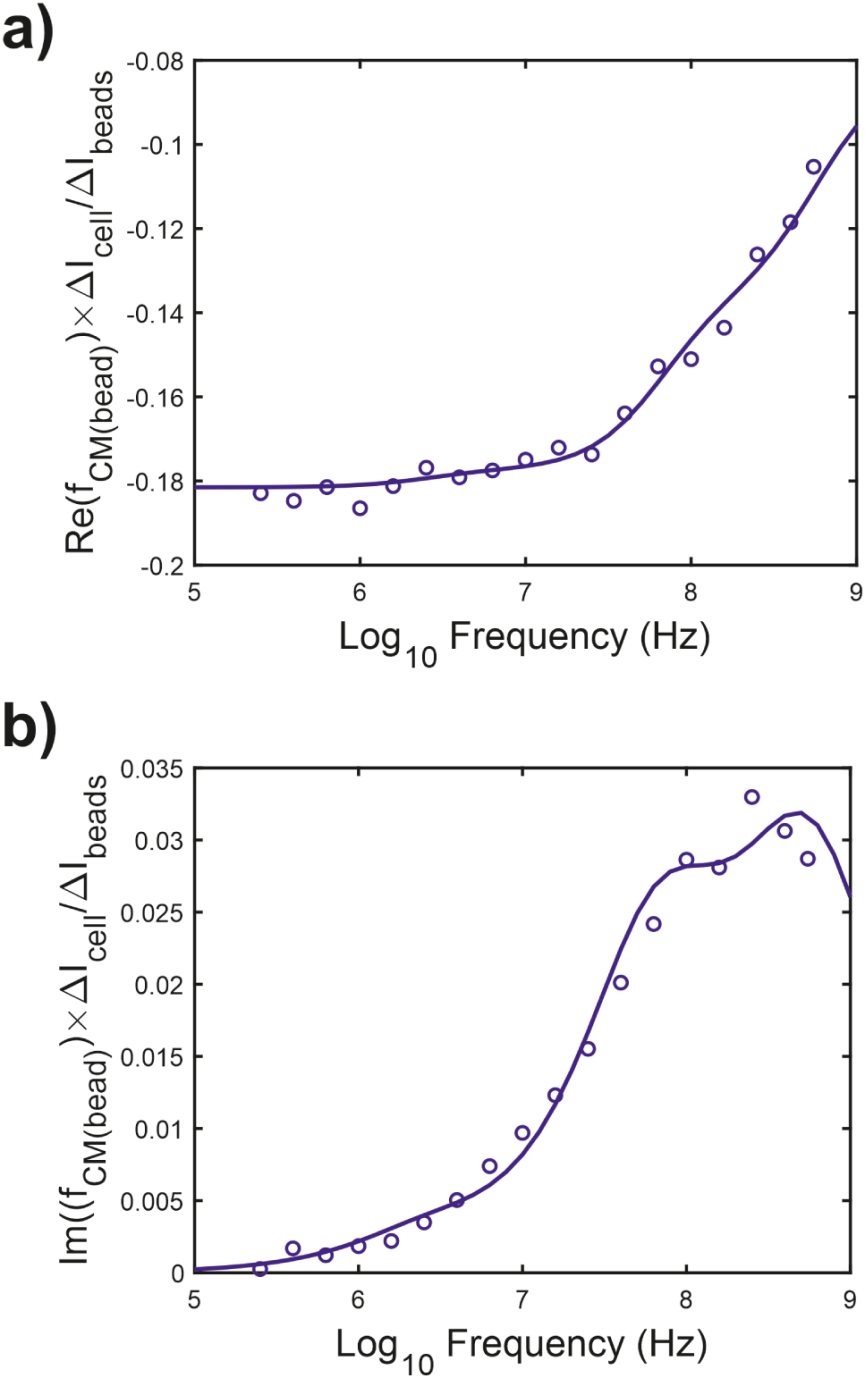
(a,b) Experimentally measured averaged impedance spectrum for a population of *Isochrysis galbana* in the f/2 medium. The *y*-axis for both plots is the mean of the real and imaginary components of the Clausius–Mossotti factor () multiplied by the ratio of the differential current measured for 10 000 cells and calibration beads in the same sample. Solid lines are the best fit for a double-shell electrical model approximating an *Isochrysis. galbana* cell.

To select two optimal frequencies for particle classification, the population-averaged impedance spectra, for example, phytoplankton species, *I. galbana*, were measured using the microcytometer. Cells were suspended in a seawater-like medium (f/2 medium). Figure 2 shows the population-averaged impedance spectra for *I. galbana* after normalization using 5 μm calibration beads; further information on the normalization procedure is detailed by Spencer et al.^21^ The solid line is the best fit for a double-shell dielectric model.^33^

The mean dielectric properties for the cells were determined by fitting a double-shell electrical model to the data using the method outlined elsewhere.^21,32^ The cell is modeled as series of concentric spherical shells, with each shell representing different cellular structures with distinct conductivity and permittivity. *I. galbana* is known to have a complex cellular ultrastructure including a thick organic scale layer outside the cell membrane approximately 100 nm thick,^34^ large internal lipid containing vesicles which can form up to 25% of the cells dry weight,^35,36^ and chloroplasts and a nucleus. A double-shell model was built with the inner core representing the cytoplasm and lipid storage vesicles,^32^ next the cell membrane, and on the outside the cell scale layer. To increase fitting accuracy, the number of free parameters was minimized by fixing the values for cell membrane permittivity based on literature values measured by electrorotation^32^ and cell scale layer thickness measured by SEM.^34^Table 1 summarizes the fitting bounds and the final fitted values. The dielectric properties which best fit the experimental data match well with those measured for *S. abundans*(32) which also has a high internal lipid content. The first dielectric relaxation is a result of polarization at the interface between the cell membrane and the suspending medium and is determined from Figure 2 as the region of maximum gradient for the real impedance values in Figure 2 a and at the peak of imaginary impedance in Figure 2b. The first dielectric relaxation for *I. galbana* was determined to be 250 MHz. This observation indicated that impedance measurements above 250 MHz would probe the internal electrical properties of particles and allow membranous cells to be differentiated from solid microplastics.

**Table 1:** Fitted Values for a Double-Shell Electrical Model of *Isochrysis galbana* Fitted to Population-Averaged Impedance Spectra

Free Parameters	Fitted Value	
inner core relative permittivity (ε_*I*_)	63	
inner core conductivity (σ_I_) (S/m)	1.12	
inner core lipid volume ratio (*L*_I_) (%)	20%	
cell membrane conductivity (σ_M_) (S/m)	5.0 × 10^–4^	
cell scale permittivity (ε_S_)	13	
cell scale conductivity (σ_M_) (S/m)	9.9 × 10^–3^	
fixed parameters	fixed value	reference
cell membrane thickness (nm)	5	(32)
cell membrane relative permittivity (*ε*_M_)	6	(32)
cell scale thickness (nm)	100	(34)

### Impedance Cytometry of Monodisperse Samples

**Figure 3:**
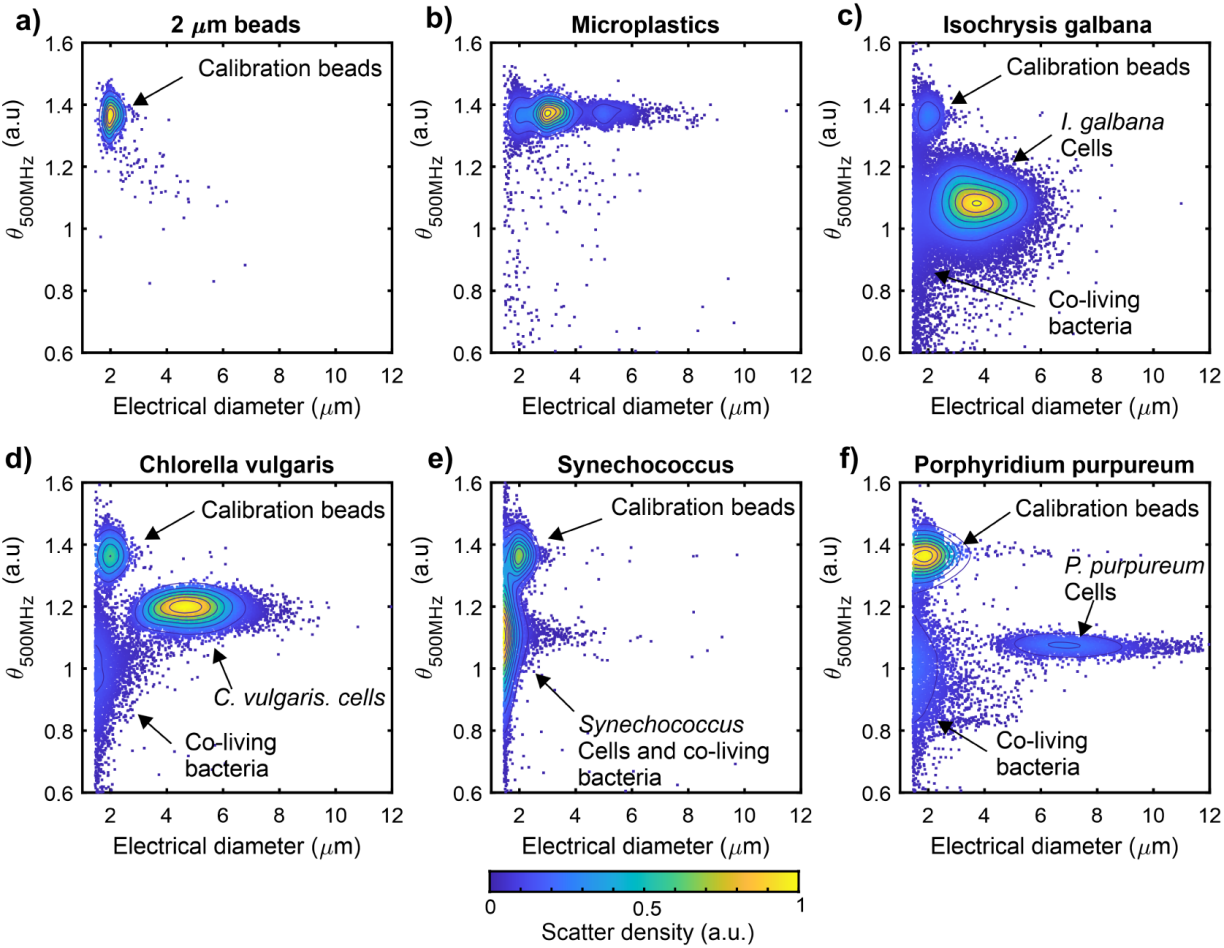
Dual-frequency impedance scatter plots for separate, monodisperse samples of (a) 2 μm polystyrene calibration beads, (b) microplastic mixture of 2, 3, and 5 μm polystyrene calibration beads and 8 μm PMMA beads, (c) *Isochrysis galbana* with 2 μm polystyrene calibration beads, (d) *Chlorella vulgaris* with 2 μm polystyrene calibration beads, (e) *Synechococcus sp*. with 2 μm polystyrene calibration beads, and (f) *Porphyridium purpureum* with 2 μm polystyrene calibration beads. On all plots, the *y-*axis is the phase measured at 500 MHz and the *x-*axis is the electrical diameter calculated from impedance measurements at 1 MHz. The density of scatter points is color-coded according to the legend, and contour lines are displayed showing lines of equal density.

Based on the data shown in Figure 2, the optimal frequencies selected for dual-frequency impedance cytometry measurements for particle classification were 1 MHz for size (volume) and 500 MHz for internal electrical properties. Dual-frequency impedance measurements were then made for monodisperse cultures of phytoplankton and microplastic controls to build a training data library for *k*-nearest neighbors classification (KNN), a supervised machine learning algorithm. Figure 3 shows contour scatter plots of the impedance phase (500 MHz) against the electrical diameter (1 MHz) for each monodisperse sample. The impedance phase at 500 MHz is displayed in arbitrary units for relative comparison. In each case, 2 μm diameter calibration beads were mixed into each sample to provide a calibration reference, and the cell data used to train the KNN model are indicated. The limit of detection for electrical diameter was 1.5 μm, and all data points below this threshold were discarded. The polystyrene and PMMA microplastic controls shown in Figure 3a,b exhibit a higher phase at 500 MHz compared to the phytoplankton control species shown in Figure 3c–f. As expected, phase measurement precision decreases with electrical diameter, as can be seen by broadening of the phase distributions as diameter decreases in all plots. Microscopy of the phytoplankton cultures showed evidence of small (<3 μm diameter) biological particles mixed in with the culture, which were positively stained for DNA, and were considered likely to be coliving bacteria and biological debris (Figure S3). The size and phase signal for the smallest phytoplankton species measured are shown in Figure 3e and overlap with signals from coliving bacteria shown in Figure 3c, d, and f. Figure 3e also shows that the population distribution for *Synechococcus* cells, which is known to have a size range from 0.5 to 1.5 μm in diameter,^37^ is significantly cropped by the detection limit of the system, and so *Synechococcus* cell numbers cannot be reliably measured on this device.

### Differentiation of Microplastics and Phytoplankton

**Figure 4:**
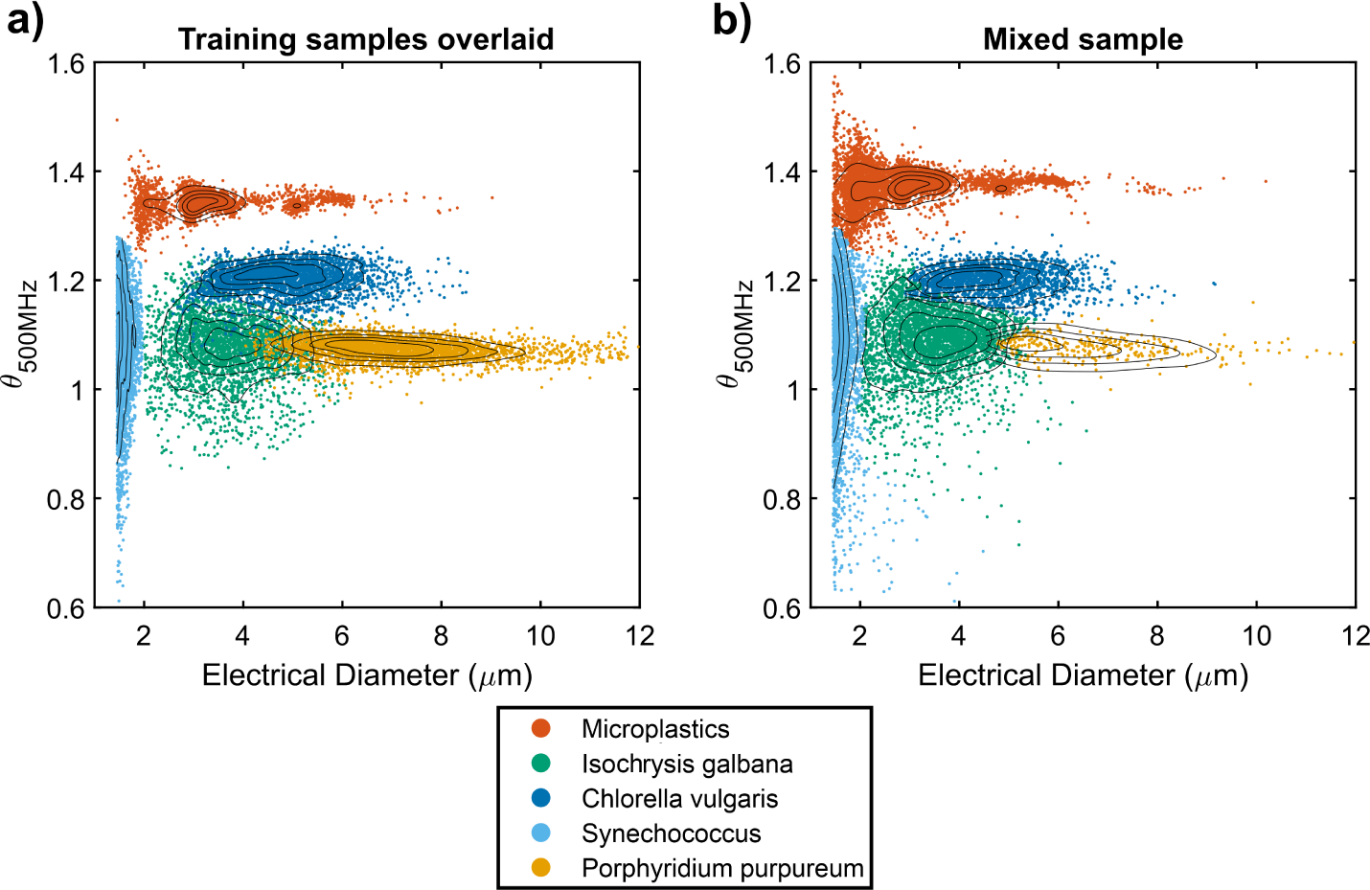
Scatter plots of dual-frequency impedance cytometry data for (a) monodisperse samples analyzed separately and overlaid on a single plot. Each sample provides a set of 3000 training data points for KNN classification for unknown particles. (b) Scatter plot of impedance cytometry data from 15000 particles in a single sample containing a mixture of the same phytoplankton and microplastics at known concentrations. Each unknown particle has been individually assigned a class by the KNN algorithm. Black contour lines indicate lines of equal particle density with increasing concentricity indicating increasing point density.

The dual-frequency impedance cytometry data from the separate microplastic and phytoplankton samples were used to train a KNN algorithm for the classification of microplastics and individual species of phytoplankton. Figure 4a shows impedance cytometry data for each monodisperse training sample overlaid on a single scatter plot. To test the KNN classification process, monodisperse samples were combined in a single mixed sample at known concentrations and analyzed on the microcytometer. The trained KNN algorithm was used to classify each detected particle into one of the training classes. Figure 4b shows the KNN classified impedance cytometry data from the mixed sample, showing a similar class distribution to the training data.

**Figure 5:**
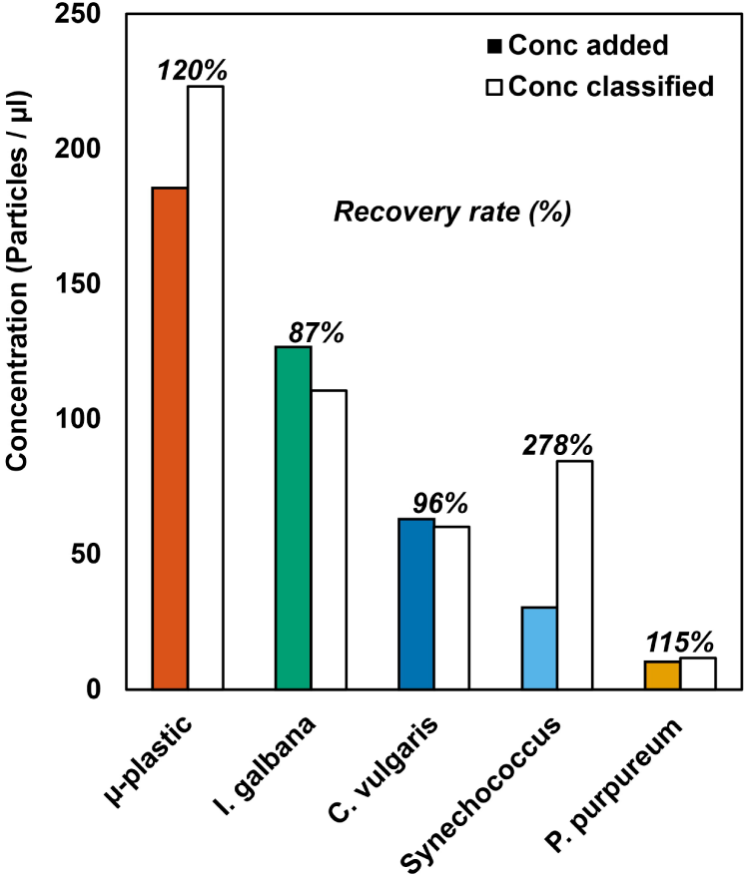
Evaluation of the KNN classification accuracy of a mixed sample of phytoplankton and microplastics. The recovery rate is the ratio of the KNN classified particle concentration against the true concentration of each particle class added to the mixed sample.

The accuracy of the KNN algorithm for particle classification was evaluated using the “recovery rate” as a metric, defined as the ratio of the KNN classified particle concentration against the true concentration for each particle class in the mixture. Figure 5 shows that classification overall was accurate to within ±20% of the true concentration for all classes except the smallest phytoplankton, *Synechococcus*. Accuracy is limited partly as the scatter distributions from *I. galbana*, *C. vulgaris*, and *P. purpureum* overlap to some extent. The additional of further independent variables such as fluorescence signals^38^ would be expected to increase the accuracy of the classification process in this case. *Synechococcus* was overestimated by a much larger margin of +178% which is attributed to the *k*-NN algorithm misclassifying bacteria as *Synechococcus*. This occurs because the *Synechococcus* size and phase signal significantly overlap with the coliving bacteria signals mixed in from the other cultures, making it appear like there are more *Synechococcus* than originally added to the mixture. The signals are likely to be similar as both bacteria and coliving bacteria are similar in size and have an external cell wall surrounding a plasma membrane. Any further differences between bacteria and *Synechococcus* in cell structure cannot be discerned within the current precision limits of the system. The signal for both *Synechococcus* and coliving bacteria also borders on the size detection limit of the device, making total counts unreliable. Reducing the channel height would be expected to improve device sensitivity, enabling smaller particles to be measured with greater precision.

**Figure 6:**
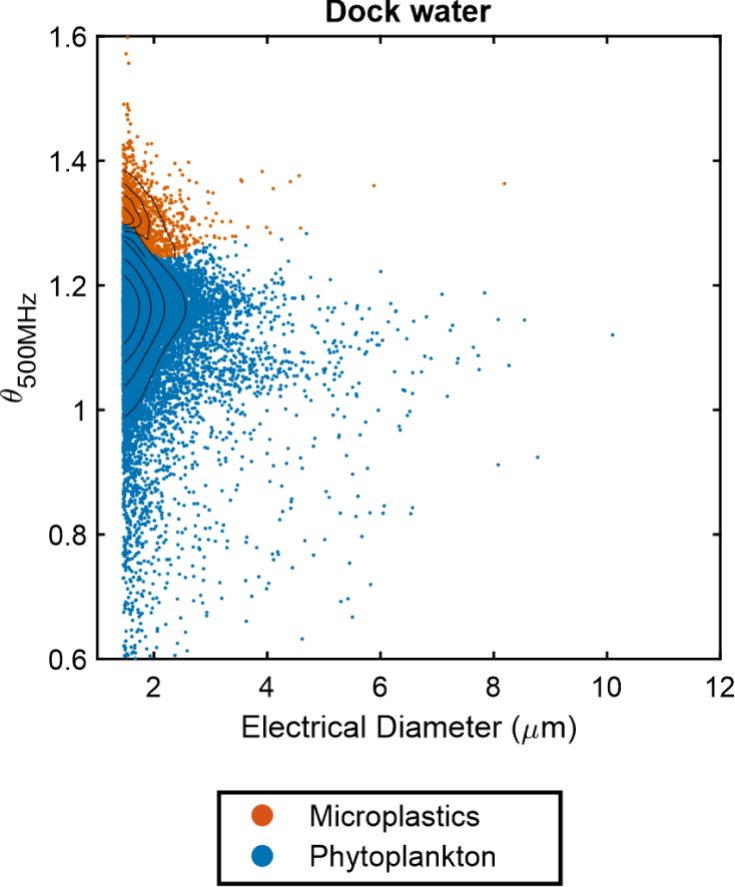
Scatter plot of dual-frequency cytometry data for a sample of dock water from the National Oceanography Centre, Southampton Docks. A binary KNN algorithm was used to classify the particles into microplastics and phytoplankton. Black contour lines indicate lines of equal particle density with increasing concentricity indicating increasing point density.

The impedance cytometer was also used to analyze a sample of seawater taken from the docks at the National Oceanography Center, Southampton, UK. To simplify the analysis, a binary KNN algorithm was used to categorize particles in the sample into either microplastics or biological particles. To achieve this, the training data for all species of phytoplankton were pooled into a single group representing biological particles. The dock water sample was also analyzed on an optical flow cytometer (Attune Nxt) to compare total particle counts (see Figures S4 and S5). Figure 6 shows the impedance cytometry data for the analysis of a dock water sample showing the classification of the sample into microplastics and phytoplankton using the binary KNN algorithm. The total particle concentration measured in the sample was 176 particles/μL for the conventional cytometer and 162 particles/μL for the impedance cytometer, showing good agreement. The concentration of each class detected by the KNN algorithm was 12 particles/μL for microplastics and 150 particles/μL for biological particles. The sample contains a high proportion of particles below 2.4 μm in diameter which are likely to be cyanobacteria and bacteria (see Figures S3 and S4). As no gold standard identification techniques for microplastics in this size range were available, these results could only be examined qualitatively as an example of an analysis of a real-world seawater sample. This analysis has identified some of the complexities associated with direct analysis of seawater with a trained machine learning algorithm. The process is currently unable to distinguish between cyanobacteria and bacteria, which could make up a high proportion of the particulate matter.

Overall, these results show that dual-frequency impedance cytometry is capable of discriminating microplastics from phytoplankton in a seawater-like medium and in measuring size distribution in the 1.5–10 μm diameter range and has some use for broad discrimination of phytoplankton species. This work used a simplified mixture of particles of known origin to demonstrate the principle of the technique in a controlled environment. Seawater samples taken from the ocean contain a complex mixture of inorganic and biological particles with multiple species of phytoplankton sometimes of unexpected origin. This analysis did not include characterization of suspended silt particles, which as solid dielectric particles may have similar high-frequency impedance properties to plastic particles. Silt can be excluded from samples by density-based separation of the sample before impedance cytometry.^39,40^ Microscopic bubbles may also exhibit impedance signals similar to those of solid particles. In our experiments, care was taken to avoid the introduction of bubbles into the sample during handling, and no significant inclusion of bubbles in the samples was detected (see Figure S5). The method could be used to analyze real seawater samples in the future by further training with additional model species or the application of unsupervised machine learning techniques. The technique also used a benchtop lock-in amplifier which can be replaced by a bespoke lock-in amplifier for deployment.

## Conclusions

We have demonstrated the use of dual-frequency impedance cytometry to discriminate between microplastics and four plankton species in a seawater-like medium at a particle size range below 10 μm. Measurement of the impedance spectrum of the model phytoplankton Isochrysis galbana showed a dielectric relaxation above 250 MHz. Based on these data, an impedance cytometry technique was developed where thousands of microplastic particles could be differentiated from phytoplankton using dual-frequency impedance measurements at 1 and 500 MHz, representative of particle size and internal electrical composition, respectively. A simple machine learning algorithm based on *k*-nearest neighbor classification allowed identification of microplastics and phytoplankton above 2 μm in size within ±20% of true concentration in a mixed culture of phytoplankton. The device was also used for a qualitative analysis of dock water identifying some of the limitations of the approach and suggesting future routes for optimization. The work presented here shows promise as a technique to characterize the smallest size fraction of phytoplankton and microplastics in the ocean. The chip-based design and all electronic sensing method with simple fluidics are highly amenable to creating a rugged deployable device which can be used to gain an insight into the particulate mixture in the ocean.

## Data Availability

Data for this publication are obtainable from 10.5258/SOTON/D2862.

## Supporting Information Available

Comparison table of impedance cytometry devices from the literature; further details on materials and methods: cross validation loss and resubstitution loss of *k*-nearest neighbor classification and photograph of impedance cytometry apparatus; further results details: microscopy analysis of monocultures used, *k*-NN classification recovery rate data table, analysis of dock water sample using a traditional flow cytometer, and bubble identification protocol (PDF)
