# Supplementary material for: Discrimination of Microplastics and Phytoplankton Using Impedance Cytometry
Source: ACS Sens. 2024 Aug 14;9(10):5206–13. doi: 10.1021/acssensors.4c01353 (PMC11519907; doi:10.1021/acssensors.4c01353)
Supplement: Supplementary file 1 — se4c01353_si_001.pdf [file se4c01353_si_001.pdf]

Supplementary information: Discrimination of microplastics and phytoplankton using impedance cytometry

Jonathan T. Butement*[a], Xiang Wang[a], Fabrizio Siracusa[b], Emily Miller[a], Katsiaryna Pabortsava[b], Matthew Mowlem[b], Daniel Spencer[a], Hywel Morgan[a]

[a] School of Electronics and Computer Science, University of Southampton, Southampton, SO17 1BJ, United Kingdom

[b] National Oceanography Centre, Southampton, SO14 3ZH, United Kingdom

Table S1: Comparison of impedance cytometry techniques from the literature with current work.

Impedance device type	Particle type	Particle size (µm)	Throughput	Impedance frequencies (MHz)	Analysis medium and conductivity	Ref
**Coplanar electrode and fluorescence microfluidic chip**	Phytoplankton and beads	1-7	10 µl/min	0.327 , 6.030	Seawater-like , 6.2 S/m	(1)
**Coplanar electrode microfluidic chip**	Phytoplankton	1.5	Unknown	0.5, 20	PBS, 1.5 S/m	(2)
**Coplanar electrode microfluidic chip**	Phytoplankton and beads	5	0.01 µl/min	0.5, 20	Seawater	(3)
**Bulk characterisation in cell culture well with commercial chip**	Phytoplankton	10-60	Unknown	0.003-10	Freshwater-like, 0.2 S/m	(4)
**Custom PCB with parallel electrodes and Flow cell**	Beads, shrimp eggs, zooplankton	200-1000	100 ml/min	0.010, 1.1	Freshwater like	(5)

**(This article) Parallel electrode microfluidic chip**	Phytoplankton, plastic beads	1.5-15	40 µl/min	1, 500	Seawater like, 2.9 S/m	NA

MATERIALS AND METHODS

K-NEAREST NEIGHBOURS CLASSIFICATION OF PARTICLES

The number of nearest neighbours (k-number) was varied between 1 and 100 and the cross-validation loss and the substitution loss for the k-NN algorithm were recorded. A K number of 11 minimised the re-substitution loss and the cross-validation loss percentage as shown in Figure S1.

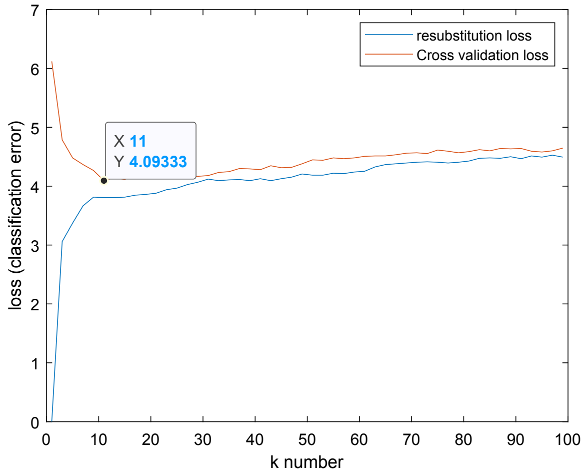

Figure S1: Classification error (%) represented by resubstitution loss and cross validation loss for a range of k-numbers.

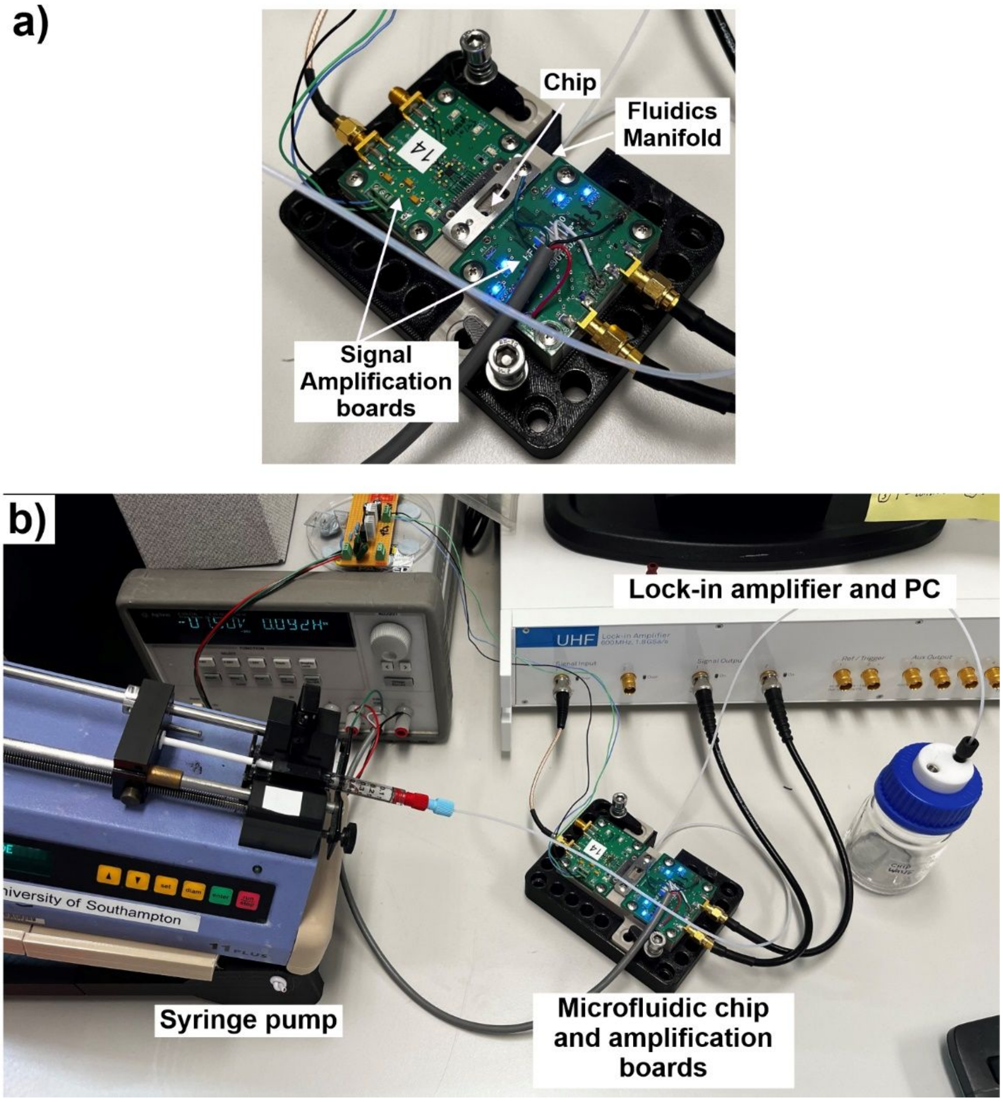

Figure S2: a) Microfluidic chip on holder with attached signal amplification boards and fluidics manifold. c) Apparatus used for impedance cytometry experiments.

RESULTS

MONOCULTURE MICROSCOPY

The composition of the monocultures was assessed by microscopy in order to inform the analysis of the impedance cytometry data. To identify bacterial contamination, the monocultures were stained with Hoechst 33342 nucleic acid stain using the manufacturers protocol. The cultures of *P.purpureum*, *I. galbana* and *C.vulgaris* were imaged on a Deltavision elite microscope imaging with a DAPI filter set for stained DNA fluorescence and a chlorophyll filter set for intrinsic phytoplankton chlorophyll. Figure S3 a), b) and c) show small cellular structures, around 1 µm or less, indicated in green (false colour representing nucleic acid stain fluorescence) which are likely to be co-living bacteria in the sample or biological debris. The bacteria and biological debris are intermixed with the phytoplankton shown in red (false colour representing Chlorophyll fluorescence). Figure S3 f) shows a fluorescence image of the Synechococcus culture imaged separately on an Olympus IXplore standard microscope using a green LED excitation source with a band pass 540/50 nm excitation filter, 555 nm long pass dichroic and 600-700 nm emission filter to image chlorophyll fluorescence emission. Due to the long exposure time on the images the moving cells appear elongated. Brightfield images shown in Figure S3 c) and e) of *C.vulgaris* and *I. galbana* were also obtained on an Olympus IXplore using a colour camera.

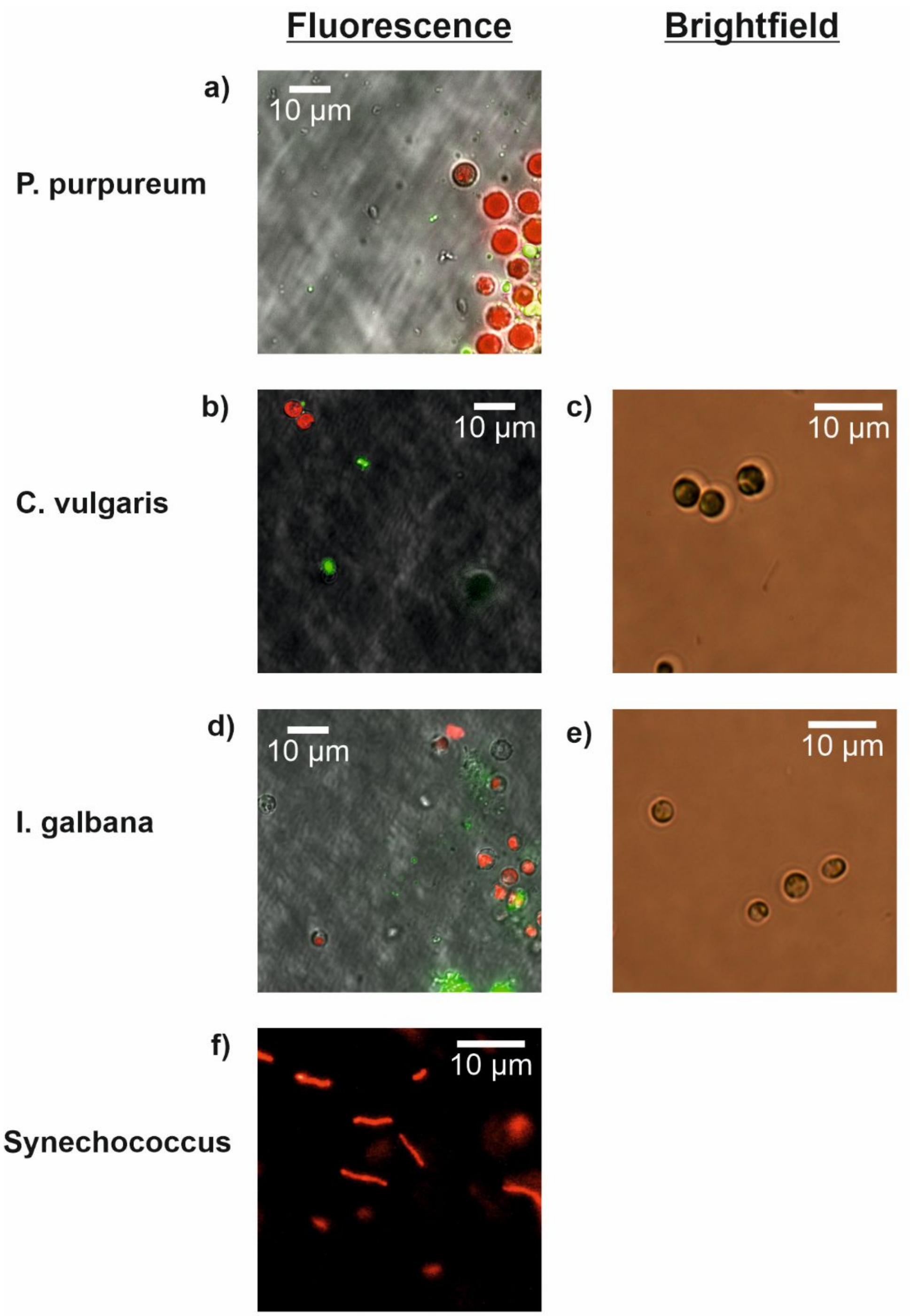

Figure S3: Microscopy photographs of monocultures. a), b) d) False colour multichannel fluorescence image showing chlorophyll fluorescence in red and Hoechst DNA stain in green. c),e) Brightfield colour images f) Fluorescence Image of Synechococcus chlorophyll fluorescence.

RECOVERY RATE

The recovery rate was defined as the ratio of the concentration of particles classified by the k-NN algorithm vs the concentration of particles added to the mixture as determined by serial dilution of stock bead concentrations and impedance cytometry of individual cultures.

Table S2: Particle recovery rate.

	Conc added (particles/µl)	Conc classified (particles/µl)	Recovery rate (%)
**Microplastics**	185.6	223.2	120
**I. galbana**	126.8	110.7	87
**C. vulgaris**	63.0	60.3	96
**Synechococcus**	30.4	84.6	278
**P. purpureum**	10.2	11.7	115
**Total**	416.0	490.4	118

FLOW CYTOMETRY OF DOCK WATER

To confirm total particle counts, the dock water sample was also analysed on a conventional flow cytometer (Attune Nxt). Figure S4 (a) shows the scatter plot of chlorophyll fluorescence (BL3-A) vs forwards scatter (FSC-A) . Figure S4 (b) shows the size distribution of particles in the dockwater compared to the forwards scatter signal from 2.4 µm calibration particles as measured on the Attune Nxt. Figure S5 shows the size distribution of particles from the dock water sample measured by impedance cytometry showing a comparable size distribution to that measured on the Attune Nxt.

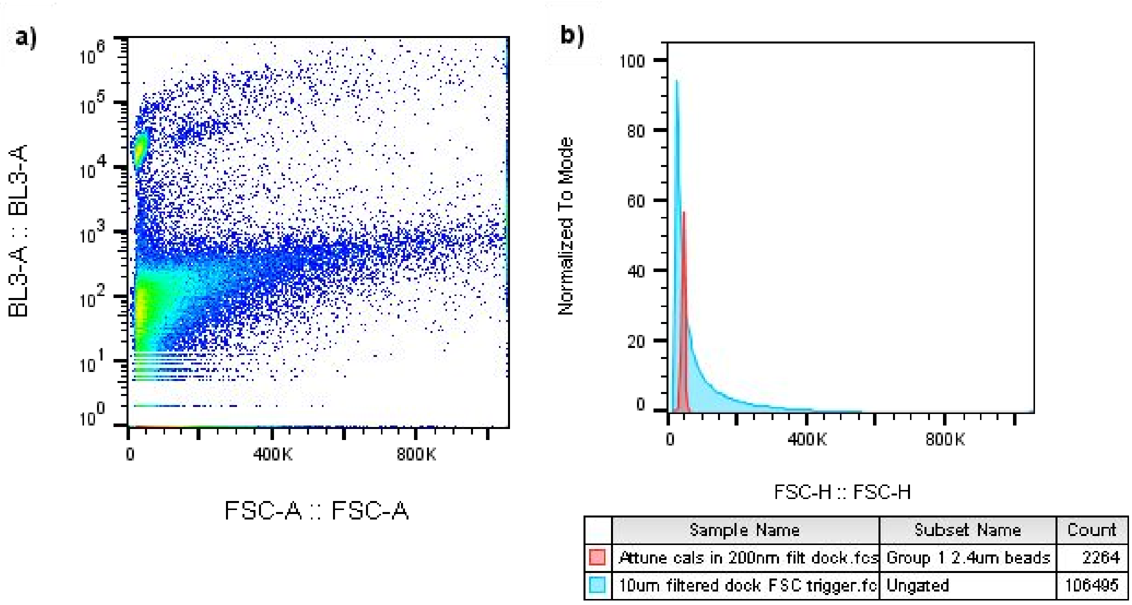

Figure S4. a) Dock water analysis on a conventional flow cytometer (Attune Nxt) showing a scatter plot of chlorophyll fluorescence (BL3-A) against forwards scatter area (FSC-A) representing particle size. b) Histogram of the size distribution of particles in the dock water sample measured using forwards scatter and calibrated using 2.4 µm diameter beads overlaid in red.

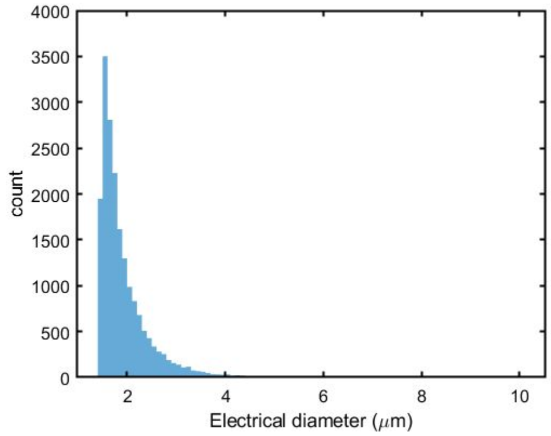

Figure S5. Size distribution of particles from the dock water sample measured on the impedance cytometer.

Table S3. Particle concentrations detected in a dock water sample by optical cytometry (Attune Nxt) and impedance cytometry. Microplastic and Biological particle concentrations were determined from the impedance cytometry data using a binary KNN model.

	**Impedance cytometer Concentration (particles/µl)**	**Attune Nxt Concentration (particles/µl)**
**Total concentration**	162	176
**Microplastics**	12	NA
**Biological particles**	150	NA

BUBBLE IDENTIFICATION

Care was taken when mixing samples to avoid inclusion of microbubbles by minimising inversion and vigorous mixing. To identify if a significant number of bubbles were included in the mixed sample the particle size distribution, as measured by impedance cytometry, of the microplastics stock sample and the microplastics in the stock solution were compared. The microplastic mixture contains 2, 3 , 5 and 8 µm calibration particles with narrow size distributions centred on their nominal diameter. Figure S6 shows that the shape of the size distributions is very similar for the stock sample in Figure S6 (a) and the mixed culture sample in Figure S6 (b). Note that the 2 µm peak in figure (b) is relatively larger as more 2 µm calibration beads were added to the mixed sample for calibration purposes. Extra peaks appearing around 4 µm and 6 µm are attributed to particles which are stuck together. The inclusion of a significant number of microbubbles would be expected to blur the individual peaks produced by the calibration particles included in the sample.

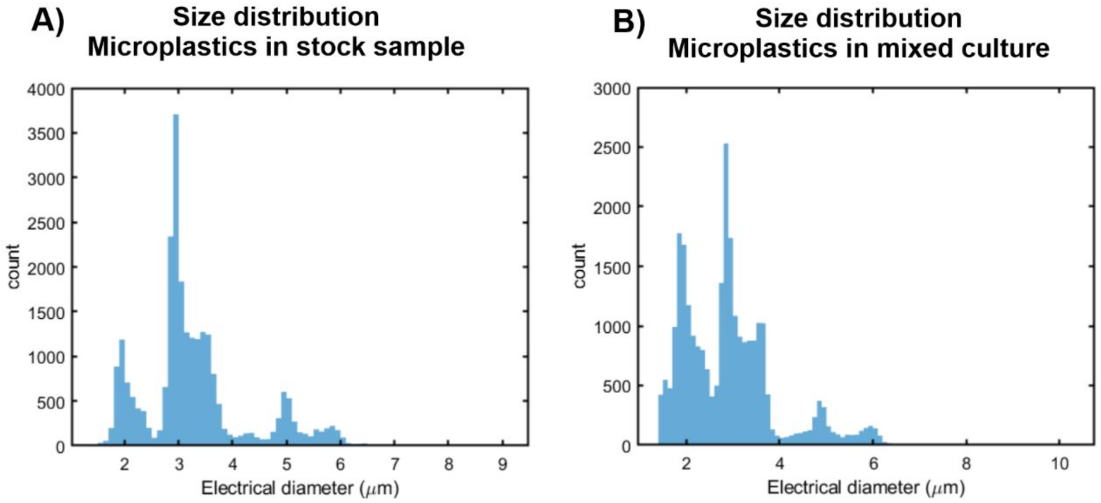

Figure S6:Microplastic size distribution measured by impedance cytometry for (a) 15 ul stock microplastics sample and (b) the microplastic component of a 60 µl mixed culture.

REFERENCES

1. Benazzi G, Holmes D, Sun T, Mowlem MC, Morgan H. Discrimination and analysis of phytoplankton using a microfluidic cytometer. IET Nanobiotechnology. 2007;1(6):94–101.

2. Sui J, Foflonker F, Bhattacharya D, Javanmard M. Electrical impedance as an indicator of microalgal cell health. Sci Rep. 2020;10(1):1–9.

3. de Bruijn DS, ter Braak PM, Van de Waal DB, Olthuis W, van den Berg A. Coccolithophore calcification studied by single-cell impedance cytometry: Towards single-cell PIC:POC measurements. Biosens Bioelectron [Internet]. 2021;173(September 2020):112808. Available from: https://doi.org/10.1016/j.bios.2020.112808

4. Jett MR, Rashed MZ, Hendricks SP, Williams SJ. Electrical characterization of phytoplankton suspensions using impedance spectroscopy. J Appl Phycol. 2021;33(3):1643–50.

5. Colson BC, Michel APM. Flow-Through Quantification of Microplastics Using Impedance Spectroscopy. ACS Sensors. 2021;6(1):238–44.
